# Effects of short- and long-term exposure to air pollution and meteorological factors on Meniere’s disease

**DOI:** 10.1038/s41598-021-95491-9

**Published:** 2021-08-09

**Authors:** Hyo Geun Choi, Chang Ho Lee, Dae Myoung Yoo, Chanyang Min, Bumjung Park, So Young Kim

**Affiliations:** 1 Hallym Data Science Laboratory, Hallym University College of Medicine, Anyang, Korea; 2 Department of Otorhinolaryngology-Head & Neck Surgery, Hallym University College of Medicine, Anyang, Korea; 3 Hallym Institute for Environmental Diseases (HIED), Chuncheon, Korea; 4 Department of Otorhinolaryngology-Head & Neck Surgery, CHA Bundang Medical Center, CHA University, Seongnam, Korea; 5 Graduate School of Public Health, Seoul National University, Seoul, Korea

**Keywords:** Neuroscience, Environmental sciences, Diseases

## Abstract

The association between air pollutants and Meniere’s disease has not been explored. The present study investigated the relationship between meteorological factors and air pollutants on Meniere’s disease. Participants, aged ≥ 40 years, of the Korean National Health Insurance Service-Health Screening Cohort were included in this study. The 7725 patients with Meniere’s disease were matched with 30,900 control participants. The moving average meteorological and air pollution data of the previous 7 days, 1 month, 3 months, and 6 months before the onset of Meniere’s disease were compared between the Meniere’s disease and control groups using conditional logistic regression analyses. Additional analyses were conducted according to age, sex, income, and residential area. Temperature range; ambient atmospheric pressure; sunshine duration; and levels of SO_2_, NO_2_, O_3_, CO, and PM_10_ for 1 month and 6 months were associated with Meniere’s disease. Adjusted ORs (odds ratios with 95% confidence interval [CI]) for 1 and 6 months of O_3_ concentration were 1.29 (95% CI 1.23–1.35) and 1.31 (95% CI 1.22–1.42), respectively; that for the 1 and 6 months of CO concentration were 3.34 (95% CI 2.39–4.68) and 4.19 (95% CI 2.79–6.30), respectively. Subgroup analyses indicated a steady relationship of O_3_ and CO concentrations with Meniere’s disease. Meteorological factors and air pollutants were associated with the rate of Meniere’s disease. In particular, CO and O_3_ concentrations were positively related to the occurrence of Meniere’s disease.

## Introduction

Air pollutants have diverse impacts on health conditions^1,2^. In addition to respiratory^3^ or cardiovascular diseases^4^, research has suggested associations between air pollutants and the nervous systems^5^. Exposure to particulate matter has been suggested to be associated with the incidence of central nervous system diseases, including Alzheimer’s and Parkinson’s disease because it is related to an increase in reactive oxygen species and neuroinflammation^5,6^. Oxidative stress and neuroinflammation may also impact the peripheral nervous system. Thus, in addition to the central nervous system, air pollution has been reported to be related to the risk of peripheral nervous system diseases, such as autonomic nervous dysfunction^7^, vestibular diseases of paroxysmal positional vertigo^8^, and Meniere’s disease^9^.

Meniere’s disease causes dizziness and has a prevalence of about 0.27% in the United Kingdom study in 2006–2010 and 0.50% in the Finland study in 2005^10,11^. It is characterized by recurrent vertigo and cochlear symptoms, such as hearing loss, tinnitus, and ear fullness^10,12,13^. Endolymphatic hydrops has been acknowledged as a pathology of Meniere’s disease, although the etiology of Meniere’s disease remains controversial^10^. The association of ambient particulate matter exposure with the occurrence of Meniere’s disease has been demonstrated in a time-series analysis study using the Poisson generalized additive model^9^. Moreover, meteorological factors of atmospheric pressure and humidity have been presumed to be associated with vertigo or Meniere’s disease^14,15^. Low atmospheric pressure and high humidity were associated with the severity of the symptom and attack of Meniere’s disease in a longitudinal study in UK^14^. However, the relationship between Meniere’s disease and multiple air pollutants, while also considering the meteorological factors, has not been investigated.

We hypothesized that meteorological factors and air pollutants might influence the occurrence of Meniere’s disease. Because the concentration of and exposure to air pollutants are influenced by meteorological factors, air pollutants should be analyzed in the context of meteorological factors. For instance, the solubility of gaseous pollutants in the atmosphere is determined by numerous meteorological factors, such as atmospheric pressure, duration of sunshine, and temperature^16,17^. Thus, this study concurrently investigated both meteorological factors and air pollutants to identify their association with Meniere’s disease. In addition, to elucidate the temporal relationship between air pollutants and Meniere’s disease, various exposure durations—1 week, 1 month, 3 months, and 6 months—to air pollutants were analyzed to evaluate their association with the occurrence of Meniere’s disease.

## Materials and methods

### Ethics

The ethics committee of Hallym University (2019-10-023) approved this study. The requirement for informed consent was waived by the Institutional Review Board of Hallym University, and all analyses complied with the regulation of the ethics committee of Hallym University.

### Study population and participant selection

We have described the Korean National Health Insurance Service-Health Screening Cohort (NHIS-HEALS), meteorological, and air pollution data in the supplement (S1 description) and in our previous studies^18–20^. The meteorological and air pollution data were assigned to the participants based on the residential address. The meteorological and air pollution data were measured by automated synoptic observing system (ASOS) in 273 place over the country hourly and manually in 94 places hourly^21^. ASOS is an automated sensor which monitored the meteorological and aviation observations in the designated area^22^.

Participants who were diagnosed with Meniere’s disease (ICD-10 codes: H810) between 2002 and 2015 were selected from 514,866 patients with 615,488,428 medical claim codes (n = 9032). To select participants who were diagnosed with Meniere's disease for the first time, we excluded those who were diagnosed with Meniere's disease between 2002 and 2003 (n = 963). The control group included patients who were not diagnosed with Meniere’s disease between 2002 and 2015 from the original population (n = 505,834). The participants who had no record since 2004 including the participants who died before 2004 (n = 1518) were excluded. Participants without audiometric examination findings (n = 16,549) were also excluded.

Participants with histories of and were treated for head trauma (S00–S09); those with available head and neck computed tomography evaluations (n = 275; n = 12,607); and those who were treated for brain tumors (C70–C72, n = 14; n = 820), disorders of the acoustic nerve (H933, n = 22; n = 120), and benign neoplasm of cranial nerves (D333, n = 23; n = 191) were excluded from the Meniere’s disease and control participants groups. A Meniere’s disease patient who did not have a record of total cholesterol was excluded (n = 1).

**Figure 1:**
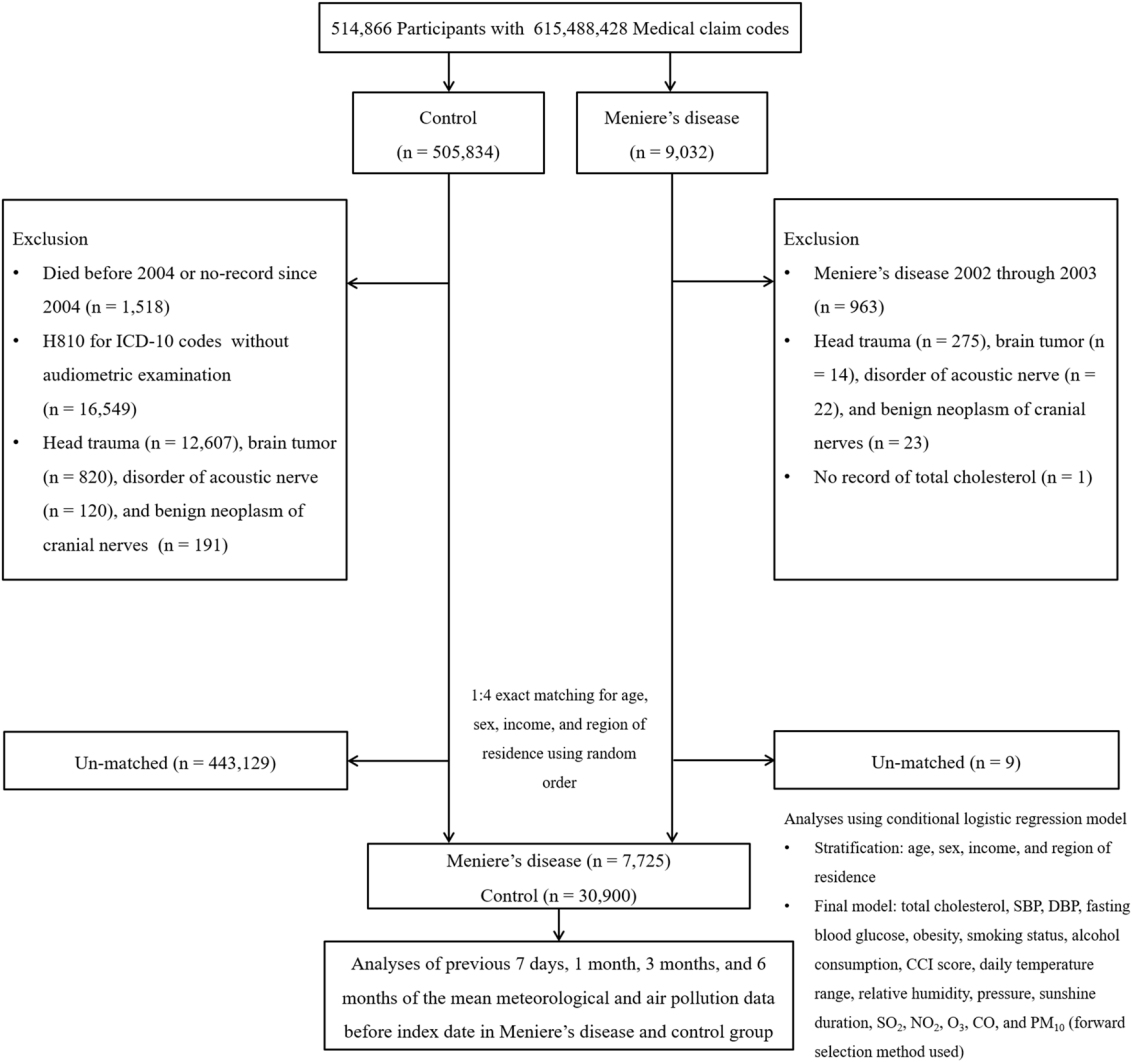
A schematic illustration of the participants' selection process that was used in the present study. Of a total of 514,866 participants, 6050 of Meniere’s disease participants were matched with 24,200 control participants for age, sex, income, and region of residence. Thereafter, the participants with Meniere’s disease and control participants were matched for the same meteorological and air pollution data before the index date.

The Meniere’s disease group was matched with the control group in a 1:4 ratio for age, sex, income, and region of residence. The date of diagnosis of Meniere’s disease was set as the index date. Using the cross-over study design with the index date, a random day in the 1-year period before the index date of the matched Meniere’s disease group was defined as the index date for the control group. Patients with Meniere’s disease who did not have enough matched control participants were excluded (n = 9); thus, 443,129 control participants were excluded during matching. In total, 7725 participants with Meniere’s disease and 30,900 control participants were included (Fig. 1).

We analyzed the meteorological and air pollution data over an average of 7 days, 1 month (30 days), 3 months (90 days), and 6 months (180 days) before the date of diagnosis of Meniere’s disease (index date, fixed exposure windows).

### Variables

#### Independent variable

Daily mean temperature (°C), daily highest temperature (°C), daily lowest temperature (°C), daily temperature range (°C) (difference between daily highest temperature and daily lowest temperature), relative humidity (%), ambient atmospheric pressure (hPa), duration of sunshine (h), rainfall (mm), SO_2_ (ppm), NO_2_ (ppm), O_3_ (ppm), CO (ppm), and PM_10_ (μg/m^3^) for a moving average of 7 days, 1 month (30 days), 3 months (90 days), and 6 months (180 days) before the index data were collected^20^. These data were gathered from Air Korea, which is managed by the Ministry of Environment in Korea^23^.

### Covariate

Age groups with 5-year intervals (40–44, 45–49, 50–54…, and 70–74 years old; 7 age groups), income groups (class 1, lowest income; to class 5, highest income), and the region of residence (urban [Seoul, Busan, Daegu, Incheon, Gwangju, Daejeon, and Ulsan] and rural [Gyeonggi, Gangwon, Chungcheongbuk, Chungcheongnam, Jeollabuk, Jeollanam, Gyeongsangbuk, Gyeongsangnam, and Jeju]) were collected^24^. Tobacco smoking (nonsmoker, past smoker, and current smoker), alcohol consumption (< times a week and ≥ 1 times a week), and obesity using body mass index (BMI; kg/m^2^) (underweight for < 18.5, normal for ≥ 18.5 to < 23, overweight for ≥ 23 to < 25, obese I for ≥ 25 to < 30, and obese II for ≥ 30)^25^ were classified based on the survey^26^. Systolic/diastolic blood pressure, fasting blood glucose, and total cholesterol levels were measured. Benign paroxysmal vertigo (H811), vestibular neuronitis (H812), and other peripheral vertigo (H813) were assigned based on the diagnostic code and clinical visits for ≥ 2 times. Comorbidities were evaluated using the Charlson Comorbidity Index (CCI) (score: 0, no comorbidities; to 29, multiple comorbidities)^27^.

#### Dependent variable

Meniere’s disease was diagnosed using ICD-10 codes (H810). Only participants who visited clinics ≥ 2 times and with available audiometric examination results (claim code: E6931–E6937, F6341–F6348) were enrolled^28,29^. The diagnostic histories of Meniere’s disease was analyzed for the associations with meteorological and air pollutant factors.

### Statistical analyses

The Meniere’s disease and control groups were compared for discrete variables using chi-square tests. The mean meteorological and air pollution data for 30 days and 180 days were compared using independent *t*-tests.

The odds ratio (OR) with 95% confidence intervals (CI) of meteorological and air pollution data for Meniere’s disease participants were calculated using crude (simple model), model 1 (adjusted for total cholesterol, SBP, DBP, fasting blood glucose, obesity, smoking status, alcohol consumption, and CCI score), and model 2 (adjusted for model 1 plus benign paroxysmal vertigo, vestibular neuronitis, other peripheral vertigo, temperature range, relative humidity, pressure, sunshine duration, SO_2_, NO_2_, O_3_, CO, and PM_10_; using the forward selection method) of conditional logistic regression. The matched variables were stratified. The results of the other days of exposure are presented in the supplement file (Table S2–S7).

Subgroup analyses were conducted according to age, sex, income, and region of residence (< 60 years old and ≥ 60 years old; men and women; low income and high income; urban and rural).

Two-tailed analyses were conducted using SAS version 9.4 (SAS Institute Inc., Cary, NC, USA). P-values < 0.05 were defined as statistically significant.

## Results

Among the meteorological and air pollution data for 30 days, mean temperature, the lowest temperature, relative humidity, ambient atmospheric pressure, rainfall, concentrations of SO_2_ (ppb), NO_2_ (ppb), O_3_ (ppb), CO (ppb), and PM_10_ (μg/m^3^) were different between Meniere's disease and control groups (all P < 0.05, Tables 1 and Table S8). For the meteorological and air pollution data for 180 days, mean temperature, the highest temperature, the lowest temperature, sunshine duration, rainfall, and the concentrations of SO_2_ (ppb), NO_2_ (ppb), O_3_ (ppb), CO (ppb), and PM_10_ (μg/m^3^) were different between the Meniere's disease group and the control group (all P < 0.05, Table 1). The distributions of BMI, smoking, CCI, benign paroxysmal vertigo, vestibular neuronitis, other peripheral vertigo, SBP, DBP, and fasting blood glucose were different between the Meniere's disease and control groups (all P < 0.05, Table 1).

**Table 1:** General characteristics of participants.

Characteristics	Total participants		
	Meniere’s disease	Control	P-value
**Age (years old, n, %)**			1.000
40–44	72 (0.9)	288 (0.9)	
45–49	470 (6.1)	1880 (6.1)	
50–54	1130 (14.6)	4520 (14.6)	
55–59	1335 (17.3)	5340 (17.3)	
60–64	1254 (16.2)	5016 (16.2)	
65–69	1231 (15.9)	4924 (15.9)	
70–74	1114 (14.4)	4456 (14.4)	
75–79	722 (9.4)	2888 (9.4)	
80–84	318 (4.1)	1272 (4.1)	
85 +	79 (1.0)	316 (1.0)	
**Sex (n, %)**			1.000
Male	2748 (35.6)	10,992 (35.6)	
Female	4977 (64.4)	19,908 (64.4)	
**Income (n, %)**			1.000
1 (lowest)	1340 (17.4)	5360 (17.4)	
2	965 (12.5)	3860 (12.5)	
3	1193 (15.4)	4772 (15.4)	
4	1603 (20.8)	6412 (20.8)	
5 (highest)	2624 (34.0)	10,496 (34.0)	
**Region of residence (n, %)**			1.000
Urban	3258 (42.2)	13,032 (42.2)	
Rural	4467 (57.8)	17,868 (57.8)	
**Obesity (BMI, kg/m**^**2**^**, n, %)**			< 0.001*
< 18.5 (underweight)	151 (2.0)	808 (2.6)	
≥ 18.5 to < 23 (normal)	2638 (34.2)	11,050 (35.8)	
≥ 23 to < 25 (overweight)	2164 (28.0)	8210 (26.6)	
≥ 25 to < 30 (obese I)	2537 (32.8)	9796 (31.7)	
≥ 30 (obese II)	235 (3.0)	1036 (3.4)	
**Smoking status (n, %)**			< 0.001*
Nonsmoker	6241 (80.8)	24,418 (79.0)	
Past smoker	812 (10.5)	2890 (9.4)	
Current smoker	672 (8.7)	3592 (11.6)	
**Alcohol consumption (n, %)**			0.574
< 1 time a week	5797 (75.0)	23,092 (74.7)	
≥ 1 time a week	1928 (25.0)	7,808 (25.3)	
**Charlson comorbidity index (n, %)**			< 0.001*
0	5160 (66.8)	22,324 (72.3)	
1	1592 (20.6)	5189 (16.8)	
2	552 (7.2)	1858 (6.0)	
3	220 (2.9)	855 (2.8)	
≥ 4	201 (2.6)	674 (2.2)	
Benign paroxysmal vertigo	2626 (34.0)	1953 (6.3)	< 0.001*
Vestibular neuronitis	841 (10.9)	441 (1.4)	< 0.001*
Other peripheral vertigo	1808 (23.4)	1400 (4.5)	< 0.001*
Total cholesterol (mg/dL, mean, SD)	200.3 (38.3)	200.0 (38.8)	0.476
SBP (mmHg, mean, SD)	126.2 (16.2)	127.0 (17.0)	< 0.001*
DBP (mmHg, mean, SD)	77.6 (10.3)	78.0 (10.7)	0.001*
Fasting blood glucose (mg/dL, mean, SD)	99.7 (25.4)	101.1 (31.2)	< 0.001*
**Meteorological factors**			
Mean temperature for 30 days (°C)	13.1 (9.4)	12.8 (9.5)	0.046*
Highest temperature for 30 days (°C)	18.2 (9.3)	18.0 (9.4)	0.057
Lowest temperature for 30 days (°C)	8.7 (9.8)	8.4 (9.9)	0.040*
Temperature range for 30 days (°C)	9.6 (2.1)	9.6 (2.1)	0.222
Relative humidity for 30 days (%)	66.1 (9.8)	65.7 (9.9)	0.005*
Ambient atmospheric pressure for 30 days (hPa)	1005.7 (7.8)	1005.9 (7.8)	0.036*
Sunshine duration for 30 days (h)	6.0 (1.3)	06.0 (1.3)	0.001*
Rainfall for 30 days (mm)	8.2 (3.7)	8.3 (4.0)	0.043*
SO_2_ for 30 days (ppb)	5.1 (1.8)	5.2 (1.8)	< 0.001*
NO_2_ for 30 days (ppb)	22.2 (9.6)	23.2 (10.0)	< 0.001*
O_3_ for 30 days (ppb)	25.6 (9.2)	24.4 (9.1)	< 0.001*
CO for 30 days (ppb)	514.9 (142.3)	522.7 (147.0)	< 0.001*
PM_10_ for 30 days (μg/m^3^)	50.2 (14.4)	51.2 (14.5)	< 0.001*
Mean temperature for 180 days (°C)	12.6 (6.2)	12.8 (6.2)	0.018*
Highest temperature for 180 days (°C)	17.8 (6.0)	18.0 (6.0)	0.012*
Lowest temperature for 180 days (°C)	8.3 (6.5)	8.4 (6.5)	0.032*
Temperature range for 180 days (°C)	9.6 (1.5)	9.6 (1.5)	0.424
Relative humidity for 180 days (%)	65.9 (6.9)	65.8 (6.9)	0.312
Ambient atmospheric pressure for 180 days (hPa)	1006.1 (6.2)	1005.9 (6.1)	0.086
Sunshine duration for 180 days (h)	6.0 (0.7)	5.9 (0.7)	< 0.001*
Rainfall for 180 days (mm)	8.2 (2.0)	8.4 (2.1)	< 0.001*
SO_2_ for 180 days (ppb)	5.1 (1.4)	5.2 (1.4)	< 0.001*
NO_2_ for 180 days (ppb)	22.4 (8.9)	23.2 (9.2)	< 0.001*
O_3_ for 180 days (ppb)	25.0 (6.8)	24.3 (6.7)	< 0.001*
CO for 180 days (ppb)	520.5 (110.7)	525.1 (113.6)	0.001*
PM_10_ for 180 days (μg/m^3^)	50.3 (9.6)	51.5 (9.8)	< 0.001*

*BMI* body mass index (kg/m^2^), *ppb* Parts per billion, *ppm* Part per million (= 1000 ppb), *SD* standard deviation.

*Chi-square test or independent T-test. Significance at P < 0.05.

The Meniere's disease group showed higher OR values for relative humidity, ambient atmospheric pressure, sunshine duration, and air pollutants of O_3_ and CO for 30 days than the control group. Adjusted OR was 1.01 (95% CI 1.00–1.01) for relative humidity; 1.01 (95% CI 1.01–1.02) for ambient atmospheric pressure; 1.08 (95% CI 1.04–1.11) for sunshine duration, 1.29 (95% CI 1.23–1.35) for O_3_, and 3.34 (95% CI 2.39–4.68) for CO (Table 2). The temperature range, SO_2_, and PM_10_ for 30 days in Meniere's disease group were associated with lower OR values than those in the control group. Adjusted OR was 0.94 (95% CI 0.92–0.97) for temperature range, 0.57 (95% CI = 0.45–0.74) for SO_2_, and 0.94 (95% CI 0.92–0.97) for PM_10_.

**Table 2:** Crude and adjusted odd ratios (95% confidence interval, CI) of the meteorological and pollution matter (mean of 30 days before index date) for Meniere’s disease.

Characteristics	Odds ratio for Meniere’s disease (95% CI)					
	Crude^†^	P-value	Model 1^†,‡^	P-value	Model 2^†,§^	P-value
Mean temperature for 30 days (10 °C)	1.03 (1.00–1.06)	0.045*	1.02 (0.99–1.05)	0.217		
Highest temperature for 30 days (10 °C)	1.03 (1.00–1.05)	0.057	1.02 (0.99–1.05)	0.279		
Lowest temperature for 30 days (10 °C)	1.03 (1.00–1.05)	0.039*	1.02 (0.99–1.05)	0.177		
Temperature range for 30 days (10 °C)	0.91 (0.79–1.04)	0.164	0.88 (0.76–1.02)	0.085	0.56 (0.44–0.71)	< 0.001*
Relative humidity for 30 days (10%)	1.04 (1.01–1.07)	0.003*	1.04 (1.01–1.07)	0.011*	1.07 (1.02–1.12)	0.003*
Ambient atmospheric pressure for 30 days (10 hPa)	0.97 (0.94–1.00)	0.034*	0.97 (0.94–1.01)	0.107	1.15 (1.10–1.21)	< 0.001*
Sunshine duration for 30 days (h)	1.03 (1.01–1.05)	0.001*	1.04 (1.02–1.06)	< 0.001*	1.08 (1.04–1.11)	< 0.001*
Rainfall for 30 days (10 mm)	0.94 (0.88–1.00)	0.052	0.91 (0.85–0.98)	0.010*		
SO_2_ for 30 days (0.01 ppm)	0.67 (0.58–0.77)	< 0.001*	0.69 (0.59–0.81)	< 0.001*	0.57 (0.45–0.74)	< 0.001*
NO_2_ for 30 days (0.1 ppm)	0.24 (0.17–0.32)	< 0.001*	0.35 (0.25–0.50)	< 0.001*		
O_3_ for 30 days (0.01 ppm)	1.17 (1.13–1.20)	< 0.001*	1.16 (1.13–1.20)	< 0.001*	1.29 (1.23–1.35)	< 0.001*
CO for 30 days (ppm)	0.68 (0.57–0.81)	< 0.001*	0.68 (0.56–0.83)	< 0.001*	3.34 (2.39–4.68)	< 0.001*
PM_10_ for 30 days (10 μg/m^3^)	0.95 (0.93–0.97)	< 0.001*	0.95 (0.94–0.97)	< 0.001*	0.94 (0.92–0.97)	< 0.001*

*CCI* Charlson comorbidity index, *DBP* diastolic blood pressure, *SBP* systolic blood pressure.

*Conditional logistic regression model, Significance at P < 0.05.

^†^Stratified model for age, sex, income, and region of residence.

^‡^A model 1 was adjusted for total cholesterol, SBP, DBP, fasting blood glucose, obesity, smoking status, alcohol consumption, and CCI score.

^§^A model 2 was adjusted for full insertion using forward selection method. The model was finally adjusted for temperature range, relative humidity, ambient atmospheric pressure, sunshine duration, SO_2_, O_3_, CO, PM_10_, obesity, smoking status, CCI score, fasting blood glucose, SBP, benign paroxysmal vertigo, vestibular neuronitis, and other peripheral vertigo.

The positive associations of Meniere's disease with ambient atmospheric pressure, sunshine duration, O_3_, and CO, and the negative associations of temperature range, SO_2_, and PM_10_ with Meniere's disease, were consistent for the 180 days of exposure (Table 3). Adjusted OR was 1.02 (95% CI 1.01–1.03) for ambient atmospheric pressure, 1.16 (95% CI 1.10–1.23) for sunshine duration, 2.03 (95% CI 1.09–3.77) for NO_2_ concentration, 1.31 (95% CI 1.22–1.42) for O_3_ concentration, and 4.19 (95% CI 2.79–6.30) for CO concentration. In contrast, the adjusted OR was 0.95 (95% CI 0.92–0.98) for temperature range, 0.50 (95% CI 0.37–0.67) for SO_2_, and 0.84 (95% CI 0.80–0.88) for PM_10_.

**Table 3:** Crude and adjusted odd ratios (95% confidence interval, CI) of the meteorological and pollution matter (mean of 180 days before index date) for Meniere’s disease.

Characteristics	Odds ratio for Meniere’s disease (95% CI)					
	Crude^†^	P-value	Model 1^†,‡^	P-value	Model 2^†,§^	P-value
Mean temperature for 180 days (10 °C)	0.95 (0.91–0.99)	0.018*	0.94 (0.90–0.98)	0.007*		
Highest temperature for 180 days (10 °C)	0.95 (0.91–0.99)	0.012*	0.93 (0.89–0.98)	0.003*		
Lowest temperature for 180 days (10 °C)	0.96 (0.92–1.00)	0.029*	0.95 (0.91–0.99)	0.015*		
Temperature range for 180 days (10 °C)	0.89 (0.71–1.11)	0.283	0.79 (0.62–1.01)	0.059	0.58 (0.42–0.80)	0.001*
Relative humidity for 180 days (10%)	1.02 (0.98–1.06)	0.264	1.02 (0.98–1.07)	0.319		
Ambient atmospheric pressure for 180 days (10 hPa)	1.04 (1.00–1.08)	0.077	1.05 (1.00–1.10)	0.035*	1.22 (1.15–1.29)	< 0.001*
Sunshine duration for 180 days (h)	1.13 (1.09–1.18)	< 0.001*	1.15 (1.11–1.20)	< 0.001*	1.16 (1.10–1.23)	< 0.001*
Rainfall for 180 days (10 mm)	0.59 (0.52–0.67)	< 0.001*	0.58 (0.51–0.67)	< 0.001*		
SO_2_ for 180 days (0.01 ppm)	0.61 (0.51–0.74)	< 0.001*	0.62 (0.50–0.77)	< 0.001*	0.50 (0.37–0.67)	< 0.001*
NO_2_ for 180 days (0.1 ppm)	0.21 (0.15–0.29)	< 0.001*	0.33 (0.22–0.48)	< 0.001*	2.03 (1.09–3.77)	0.026*
O_3_ for 180 days (0.01 ppm)	1.21 (1.17–1.27)	< 0.001*	1.20 (1.15–1.26)	< 0.001*	1.31 (1.22–1.42)	< 0.001*
CO for 180 days (ppm)	0.68 (0.54–0.86)	0.001*	0.65 (0.51–0.84)	0.001*	4.19 (2.79–6.30)	< 0.001*
PM_10_ for 180 days (10 μg/m^3^)	0.88 (0.86–0.91)	< 0.001*	0.88 (0.86–0.91)	< 0.001*	0.84 (0.80–0.88)	< 0.001*

*CCI* Charlson comorbidity index, *DBP* diastolic blood pressure, *SBP* systolic blood pressure.

*Conditional logistic regression model, Significance at P < 0.05.

^†^Stratified model for age, sex, income, and region of residence.

^‡^A model 1 was adjusted for total cholesterol, SBP, DBP, fasting blood glucose, obesity, smoking status, alcohol consumption, and CCI score.

^§^A model 2 was adjusted for full insertion using forward selection method. The model was finally adjusted for temperature range, ambient atmospheric pressure, sunshine duration, SO_2_, NO_2_, O_3_, CO, PM_10_, obesity, smoking status, CCI score, fasting blood glucose, SBP, benign paroxysmal vertigo, vestibular neuronitis, and other peripheral vertigo.

**Figure 2:**
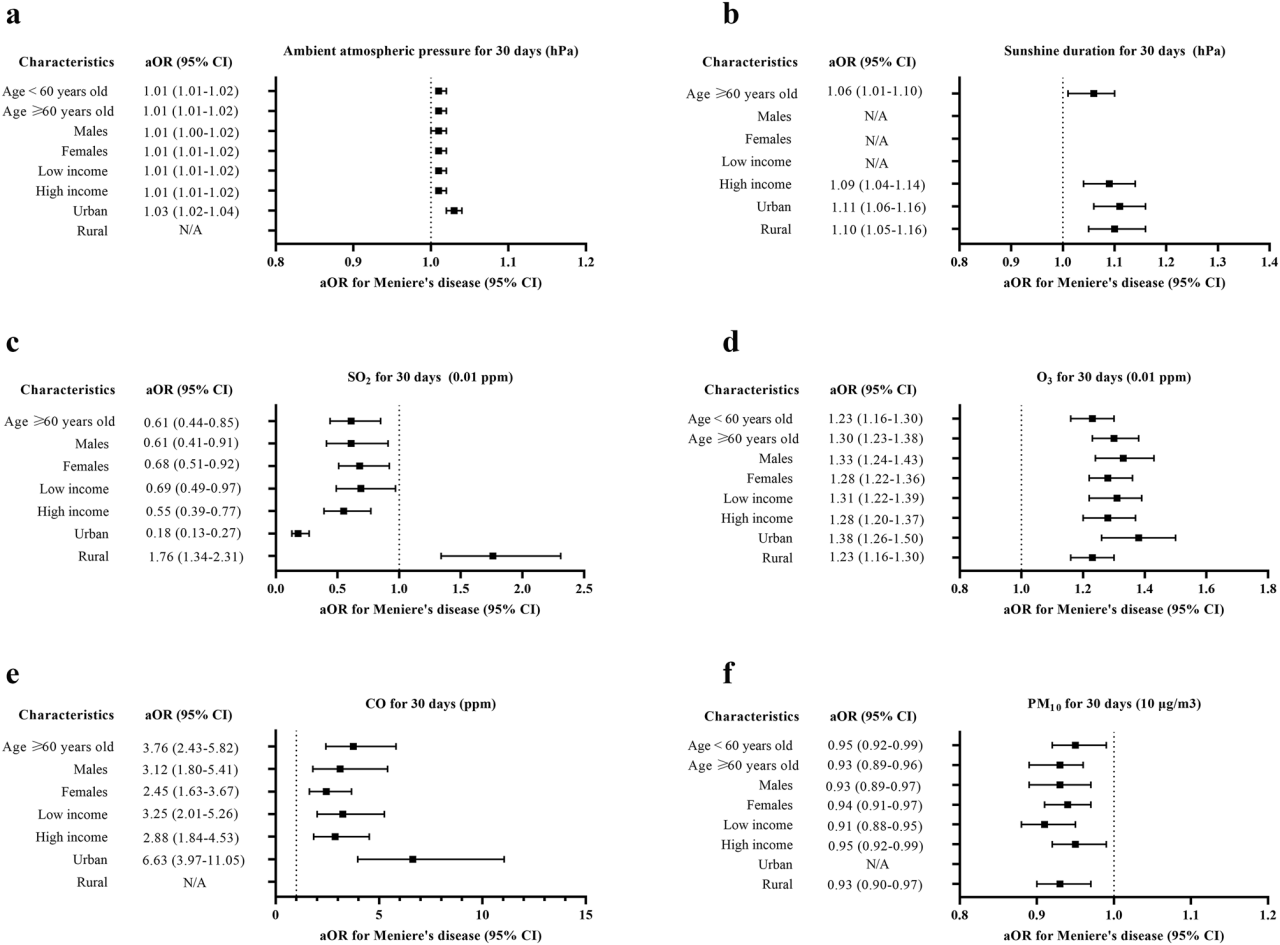
Subgroup analyses of odd ratios (95% confidence interval [CI]) of (**a**) ambient atmospheric pressure for 30 days (hPa) (high odds ratios in the Meniere’s disease group of all analyzed subgroup except for rural subgroup), (**b**) sunshine duration for 30 days (h) (high odds ratios in the Meniere’s disease group of ≥ 60 years old, high income, and urban subgroups) (**c**), SO_2_ for 30 days (0.01 ppm) (high odds ratios in the Meniere’s disease group of ≥ 60 years old and rural subgroups), (**d**) O_3_ for 30 days (0.01 ppm) (high odds ratios in the Meniere’s disease group of all subgroups), (**e**) CO for 30 days (ppm) (high odds ratios in the Meniere’s disease group of ≥ 60 years old, males, females, low income, high income, and urban subgroups), (**f**) and PM_10_ for 30 days (10 μg/m^3^) (low odds ratios in the Meniere’s disease group of all subgroups except for urban subgroup). (NA: not available).

**Figure 3:**
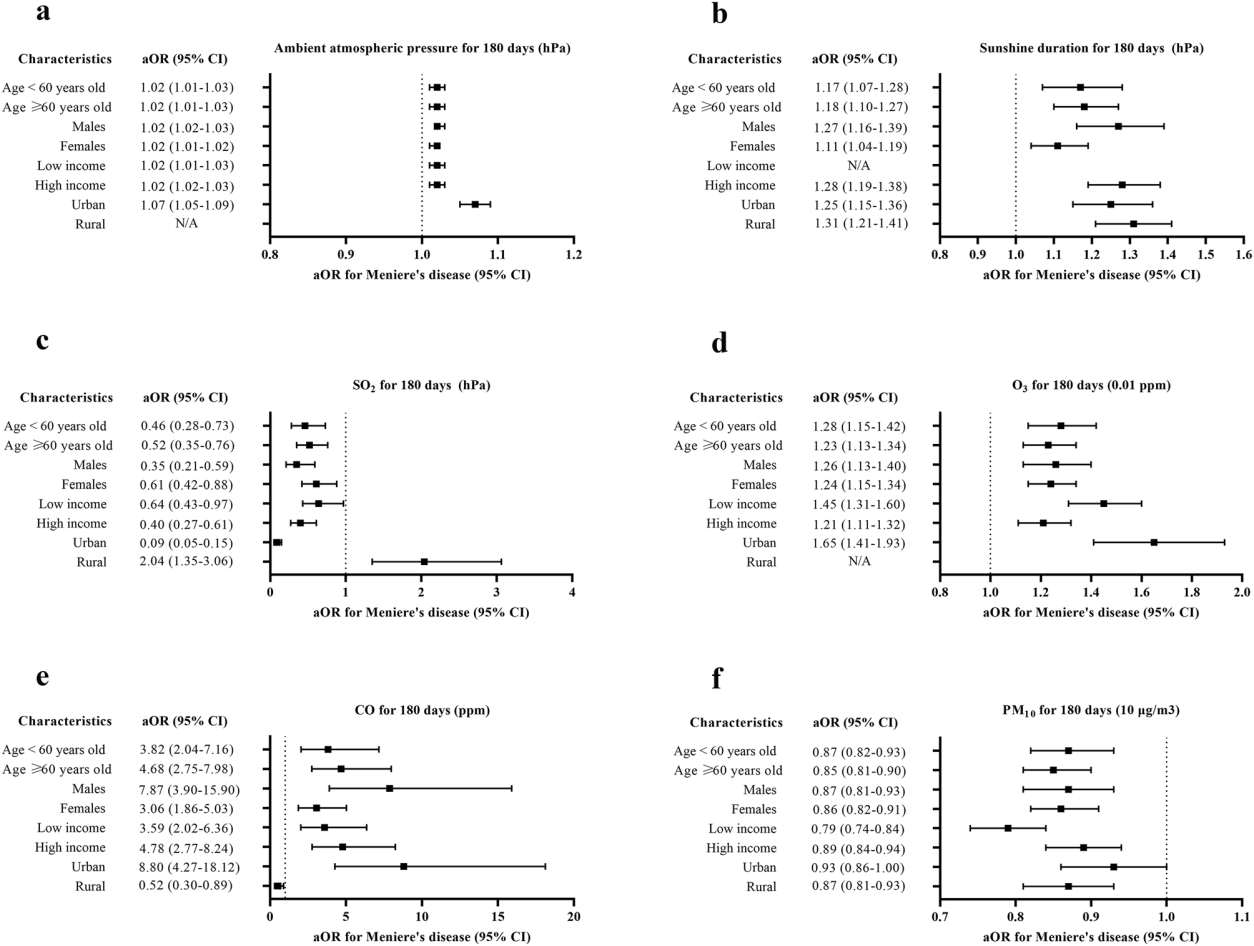
Subgroup analyses of odd ratios (95% confidence interval [CI]) of (**a**) ambient atmospheric pressure for 180 days (hPa) (high odds ratios in the Meniere’s disease group of all analyzed subgroup except for rural subgroup), (**b**) sunshine duration for 180 days (h) (high odds ratios in the Meniere’s disease group of all subgroups except for low income subgroup) (**c**), SO_2_ for 180 days (0.01 ppm) (low odds ratios in the Meniere’s disease group of all subgroups except for rural subgroup), (**d**) O_3_ for 180 days (0.01 ppm) (high odds ratios in the Meniere’s disease group of all subgroups except for rural subgroup), (**e**) CO for 180 days (ppm) (high odds ratios in the Meniere’s disease group of all subgroups except for rural subgroup), (**f**) and PM_10_ for 180 days (10 μg/m^3^) (low odds ratios in the Meniere’s disease group of all subgroups except for urban subgroup). (NA: not available).

These relationships between meteorological factors and air pollutants with Meniere's disease were valid in the subgroups of age, sex, income, and region of residence for both 30 and 180 days of exposure (Figs. 2, 3, Tables S2 and S3). The 7 days and 90 days exposure also showed a relationship between relative humidity, ambient atmospheric pressure, sunshine duration, O_3_, and CO, with an overall higher OR values for Meniere's disease; this association was also observed in the age, sex, income, and region of residence subgroups (Tables S4–S7).

## Discussion

Higher exposures to CO and O_3_ were associated with the occurrence of Meniere’s disease. Among the meteorological factors, ambient atmospheric pressure and relative humidity were positively related with the occurrence of Meniere’s disease, whereas temperature range showed a negative association. These associations between air pollutants and meteorological factors were maintained for 7, 30, 90, and 180 days of exposure.

Exposure to CO had the highest OR values for the histories (incidence) of Meniere’s disease in the present study. A few previous studies have reported the risk of hearing loss and vertigo to be associated with chronic CO exposure^30–32^. The main source of CO is the incomplete combustion of hydrocarbons from carbon-based fuels^33^. The increase in free radical synthesis and the decrease in blood oxygen levels due to the high affinity of CO to hemoglobin (carboxyhemoglobin) were presumed to be the pathophysiology of inner ear dysfunction following prolonged CO exposure in animal studies^34,35^. Although the exposure concentration of CO was not as high as to occur carboxyhemoglobin in animal studies, since the inner ear is supplied by the end artery and is vulnerable to ischemic injury, reduced blood supply due to carboxyhemoglobin may induce inner ear dysfunction. Elevated levels of oxidative stress may result in neuroinflammation and neuronal necrosis^36^. Moreover, it has been reported that CO can function as a neurotransmitter and induce neurotoxicity in conditions of high exposure^36,37^. In summary, the oxidative stress and neurotoxic effects could contribute to the increased rate of Meniere’s disease related to CO exposure.

Exposure to O_3_ was associated with a high rate of Meniere’s disease in this study. A few studies have reported the association between O_3_ and Meniere’s disease, vertigo, or other inner ear diseases. O_3_ exposure was associated with the risk of respiratory diseases, such as acute respiratory distress syndrome^38^; cardiovascular diseases, such as hypertension^39^; and mortality in the elderly^40^. Moreover, because the inner ear is vulnerable to ischemic injury, the cardiovascular compromise related to O_3_ exposure could influence the risk of Meniere’s disease. In addition, the increased risk of autoimmunity by air pollutants could mediate the occurrence of Meniere’s disease. Through binding with aryl hydrocarbon reports, air pollutants can modulate activities of T helper 17 cells and regulatory T cells, in that cause autoimmune responses^41^. Autoimmune diseases have been known to contribute to the development of Meniere’s disease^42–44^. Compared to general population, the patients with Meniere’s disease showed higher prevalence for rheumatoid arthritis, systemic lupus erythematosus, and ankylosing spondylitis (1.39%, 0.87%, and 0.70%, respectively)^42^. A case–control study demonstrated the higher prevalence of immune genotype associated with autoimmune diseases in the patients with Meniere’s disease^43^. Thus, the common pathophysiology of autoimmune could link the association between air pollution and Meniere’s disease.

Meteorological factors, including ambient atmospheric pressure, relative humidity, and temperature range, were related to the risk of Meniere’s disease in this study. Two previous studies have also demonstrated the associations between meteorological factors of atmospheric pressure and humidity with the risk of Meniere’s disease^14,45^. The risk of Meniere’s disease attack was 1.30 times higher at an atmospheric pressure below 1013 hectopascals (95% CI 1.10–1.54)^14^. In addition, a high humidity (above 90%) was related to a 1.26-fold higher incidence of Meniere’s disease (95% CI 1.06–1.49)^14^. The potential impacts of changes in atmospheric pressure on endolymphatic pressure via the middle ear have been postulated^46^. In addition, an experimental study suggested the existence of an atmospheric pressure sensor in the vestibular system^47^. Temperature changes are associated with fluctuations in atmospheric pressure and seasons. In addition, the variabilities of temperature or atmospheric pressure could act as a stressor and induce physiological responses^48^.

To the best of our knowledge, this is the largest population data analyzed to identify the impacts of meteorological and air pollutants factors on Meniere’s disease. Owing to the large study population, we could include enough control group participants to matched for age, sex, income, and region of residence. Past medical histories and lifestyle factors, such as obesity, smoking, and alcohol consumption, were different between control and Meniere’s group, in that these variables were adjusted to attenuate the possible confounding effects. Moreover, multiple exposure durations were analyzed for their association with Meniere’s disease. However, the results of vestibular function tests and pure tone audiometry tests were not available because this study was based on health claims data. In addition, undiagnosed or subclinical cases could not be included. For meteorological and air pollutants, indoor exposures could not be measured. Although many possible confounders were adjusted, the impacts of remaining potential confounders, such as sleep time and stress level, could not be totally excluded in the current study. Because this study was an observation study, the causality between air pollutants and Meniere’s disease could not be determined. Lastly, because the study population was confined to Korea, ethnic or regional differences could exist for other populations.

## Conclusion

Both meteorological and air pollutants were related to the occurrence of Meniere’s disease. In particular, increased exposure to O_3_ and CO was associated with a higher incidence of Meniere’s disease.

## Supplementary Information

Supplementary Information.

## Data Availability

Releasing of the data by the researcher is not allowed legally. All of data are available from the database of National health Insurance Sharing Service (NHISS, https://nhiss.nhis.or.kr/). NHISS allows all of this data for the any researcher who promises to follow the research ethics with some cost. If you want to access the data of this article, you could download it from the website after promising to follow the research ethics.

## Abbreviations

NHIS-HEALS: Korean National Health Insurance Service-Health Screening Cohort
BMI: Body mass index
CCI: Charlson Comorbidity Index
OR: Odds ratio
CI: Confidence intervals
SD: Standard deviation

## References

[1] Yang D, Yang X, Deng F, Guo X (2017). Ambient air pollution and biomarkers of health effect. Adv. Exp. Med. Biol..

[2] Park M, Lee JS, Park MK (2019). The effects of air pollutants on the prevalence of common ear, nose, and throat diseases in south Korea: A National Population-Based Study. Clin. Exp. Otorhinolaryngol..

[3] Guan WJ, Zheng XY, Chung KF, Zhong NS (2016). Impact of air pollution on the burden of chronic respiratory diseases in China: Time for urgent action. Lancet.

[4] Pope CA (2009). Cardiovascular mortality and exposure to airborne fine particulate matter and cigarette smoke: Shape of the exposure-response relationship. Circulation.

[5] Babadjouni RM (2017). Clinical effects of air pollution on the central nervous system; A review. J. Clin. Neurosci..

[6] Block ML, Calderon-Garciduenas L (2009). Air pollution: Mechanisms of neuroinflammation and CNS disease. Trends Neurosci..

[7] Chen JC (2006). Personal coronary risk profiles modify autonomic nervous system responses to air pollution. J. Occup. Environ. Med..

[8] Mariani P, Pelagatti M, Hahn A, Alpini D (2008). Epidemiology of paroxysmal positioning vertigo: Correlation with seasons, climate, and pollution. Int. Tinnitus J..

[9] Han C, Lim YH, Jung K, Hong YC (2017). Association between ambient particulate matter and disorders of vestibular function. Environ. Res..

[10] Nakashima T (2016). Meniere's disease. Nat. Rev. Dis. Primers.

[11] Havia M, Kentala E, Pyykko I (2005). Prevalence of Meniere's disease in general population of Southern Finland. Otolaryngol. Head Neck Surg..

[12] Lopez-Escamez JA (2015). Diagnostic criteria for Meniere's disease. J. Vestib. Res..

[13] Kim CH, Shin JE, Yoo MH, Park HJ (2019). Direction-changing and direction-fixed positional nystagmus in patients with vestibular neuritis and Meniere disease. Clin. Exp. Otorhinolaryngol..

[14] Schmidt W (2017). The weather and Meniere's Disease: A longitudinal analysis in the UK. Otol. Neurotol..

[15] Pereira AB (2015). Seasonality of dizziness and vertigo in a tropical region. Chronobiol. Int..

[16] Wang R (2019). Cross-sectional associations between long-term exposure to particulate matter and depression in China: The mediating effects of sunlight, physical activity, and neighborly reciprocity. J. Affect. Disord..

[17] Deug-Soo Kim JJ (2016). Characteristics in atmospheric chemistry between NO, NO2 and O3 at an urban site during MAPS (Megacity Air Pollution Study)-Seoul, Korea. J. Korean Soc. Atmos. Environ..

[18] Kim SY, Min C, Oh DJ, Choi HG (2019). Tobacco smoking and alcohol consumption are related to benign parotid tumor: A nested case-control study using a National Health Screening Cohort. Clin. Exp. Otorhinolaryngol..

[19] Choi HG, Min C, Kim SY (2019). Air pollution increases the risk of SSNHL: A nested case-control study using meteorological data and national sample cohort data. Sci. Rep..

[20] Kim SY, Kong IG, Min C, Choi HG (2019). Association of air pollution with increased risk of peritonsillar abscess formation. JAMA Otolaryngol. Head Neck Surg..

[21] Service, K. W. D. *Open MET Data Portal*. https://data.kma.go.kr/resources/html/en/aowdp.html.

[22] *Automated Surface Observing System (ASOS)*. <https://www.ncdc.noaa.gov/data-access/land-based-station-data/land-based-datasets/automated-surface-observing-system-asos.

[23] *Air Korea*. http://www.airkorea.or.kr/.

[24] Kim SY, Min C, Oh DJ, Choi HG (2020). Bidirectional association between GERD and asthma: two longitudinal follow-up studies using a national sample cohort. J. Allergy Clin. Immunol. Pract..

[25] WHO/IASO/IOTR. The Asia-Pacific Perespective: Redefining Obesity and its Treatment. *Health Communications Australia Pty Ltd* (2000).

[26] Kim SY, Oh DJ, Park B, Choi HG (2020). Bell's palsy and obesity, alcohol consumption and smoking: A nested case-control study using a national health screening cohort. Sci. Rep..

[27] Quan H (2011). Updating and validating the Charlson comorbidity index and score for risk adjustment in hospital discharge abstracts using data from 6 countries. Am. J. Epidemiol..

[28] Kim SY, Lee CH, Min C, Park IS, Choi HG (2020). Bidirectional analysis of the association between Meniere's disease and depression: Two longitudinal follow-up studies using a national sample cohort. Clin. Otolaryngol..

[29] Kim SY (2020). Association between Meniere's disease and thyroid diseases: A nested case-control study. Sci. Rep..

[30] Mehrparvar AH (2013). Hearing loss due to carbon monoxide poisoning. Case Rep. Otolaryngol..

[31] Seale B, Ahanger S, Hari C (2018). Subacute carbon monoxide poisoning presenting as vertigo and fluctuating low frequency hearing loss. J. Surg. Case Rep..

[32] Lakhani R, Bleach N (2010). Carbon monoxide poisoning: An unusual cause of dizziness. J. Laryngol. Otol..

[33] Weaver LK (2009). Clinical practice: Carbon monoxide poisoning. N. Engl. J. Med..

[34] Fechter LD, Liu Y, Pearce TA (1997). Cochlear protection from carbon monoxide exposure by free radical blockers in the guinea pig. Toxicol. Appl. Pharmacol..

[35] Fechter LD, Thorne PR, Nuttall AL (1987). Effects of carbon monoxide on cochlear electrophysiology and blood flow. Hear Res..

[36] Mannaioni PF, Vannacci A, Masini E (2006). Carbon monoxide: The bad and the good side of the coin, from neuronal death to anti-inflammatory activity. Inflamm. Res..

[37] Boehning D (2003). Carbon monoxide neurotransmission activated by CK2 phosphorylation of heme oxygenase-2. Neuron.

[38] Ware LB (2016). Long-term ozone exposure increases the risk of developing the acute respiratory distress syndrome. Am. J. Respir. Crit. Care Med..

[39] Cai Y (2016). Associations of short-term and long-term exposure to ambient air pollutants with hypertension: A systematic review and meta-analysis. Hypertension.

[40] Di Q (2017). Association of short-term exposure to air pollution with mortality in older adults. JAMA.

[41] Zhao CN (2019). Emerging role of air pollution in autoimmune diseases. Autoimmun. Rev..

[42] Gazquez I (2011). High prevalence of systemic autoimmune diseases in patients with Meniere's disease. PLoS ONE.

[43] Frejo L (2017). Regulation of Fn14 receptor and NF-kappaB underlies inflammation in Meniere's disease. Front. Immunol..

[44] Gazquez I, Requena T, Espinosa JM, Batuecas A, Lopez-Escamez JA (2012). Genetic and clinical heterogeneity in Meniere's disease. Autoimmun. Rev..

[45] Kim MH, Cheon C (2020). Epidemiology and seasonal variation of Meniere's disease: Data from a population-based study. Audiol. Neurootol..

[46] Merchant SN, Adams JC, Nadol JB (2005). Pathophysiology of Meniere's syndrome: Are symptoms caused by endolymphatic hydrops?. Otol. Neurotol..

[47] Sato J (2003). Weather change and pain: A behavioral animal study of the influences of simulated meteorological changes on chronic pain. Int. J. Biometeorol..

[48] Ouchi Y, Tanizawa H, Shiraishi J-I, Cockrem JF, Chowdhury VS, Bungo T (2020). Repeated thermal conditioning during the neonatal period affects behavioral and physiological responses to acute heat stress in chicks. J. Therm. Biol..

