# Supplementary material for: Effects of short- and long-term exposure to air pollution and meteorological factors on Meniere’s disease
Source: Sci Rep. 2021 Aug 9;11:16063. doi: 10.1038/s41598-021-95491-9 (PMC8352931; doi:10.1038/s41598-021-95491-9)
Supplement: Supplementary file 1 — Supplementary Information. [file 41598_2021_95491_MOESM1_ESM.pdf]

Effects of short- and long-term exposure to air pollution and meteorological factors on Meniere's disease

Hyo Geun Choi[1,2,3], Chang Ho Lee[4], Dae Myoung Yoo[1], Chanyang Min[1,5], Bumjung Park[2], So Young Kim[4*]

[1]Hallym Data Science Laboratory, Hallym University College of Medicine, Anyang, Korea

[2]Department of Otorhinolaryngology-Head & Neck Surgery, Hallym University College of Medicine, Anyang, Korea

[3]Hallym Institute for Environmental Diseases (HIED), Chuncheon, Korea

[4]Department of Otorhinolaryngology-Head & Neck Surgery, CHA Bundang Medical Center, CHA University, Seongnam, Korea

[5]Graduate School of Public Health, Seoul National University, Seoul, Korea

Running title: Air pollution and Meniere's disease

*** correspondence:** sossi81@hanmail.net

S1 Description Study Population and Data Collection

This national cohort study relied on data from the Korean National Health Insurance Service-Health Screening Cohort (NHIS-HEALS) (Lee et al. 2017). The Korean National Health Insurance Service (NHIS) randomly selects approximately 10% of individuals who underwent health examinations from 2002 to 2003 (n = ~515,000) directly from the entire population (n = ~5,150,000). Age and sex specific distributions of the cohort population are described online . The details of the methods used to perform these procedures are provided by the National Health Insurance Sharing Service (http://nhiss.nhis.or.kr/).

All insured Koreans who are at least 40 years old and their dependents undergo no-cost biannual health examinations (Song et al. 2014). Each examinee must complete a standard questionnaire in for this health screening program (Song et al. 2014). Because all Korean citizens are recognized by a 13-digit resident registration number from birth to death, exact population statistics can be determined using this database. It is mandatory for all Koreans to enroll in the NHIS. All Korean hospitals and clinics use the 13-digit resident registration number to register individual patients in the medical insurance system. Therefore, the risk of overlapping medical records is minimal, even if a patient moves from one place to another. Moreover, all medical treatments in Korea can be tracked without exception using the Korean Health Insurance Review & Assessment (HIRA) system. In Korea, providing a notice of death to an administrative entity is legally required before a funeral can be held, and the cause and date of death are recorded by medical doctors on a death certificate.

This cohort database includes (i) personal information, (ii) health insurance claim codes (procedures and prescriptions), (iii) diagnostic codes using the International Classification of Disease-10 (ICD-10), (iv) death records from the Korean National Statistical Office (using the Korean Standard Classification of disease), (v) socioeconomic data (residence and income), (vi) medical examination data (vii) health examination data (body mass index

[BMI], drinking and smoking habits, blood pressure, urinalysis, hemoglobin, fasting glucose, lipid parameters, creatinine, and liver enzymes) for each participant over the period from 2002 to 2013 (Song et al. 2014; http://nhiss.nhis.or.kr/).

Meteorological Data

Temperature (˚C), Relative humidity (%), were Spot atmospheric pressure (hPa) data were obtained from the meteorological administration. It was measured by automated synoptic observing system (ASOS) and manually in 94 places hourly. Quality was controlled following quality inspection manual (https://data.kma.go.kr/cmmn/main.do).

SO_2 (ppm), NO_2 (ppm), O_3 (ppm), CO (ppm), and PM_{10} (μg/m^3) data were obtained by the ministry of environment. It was measured by ASOS in 273 place over the country hourly. Quality was controlled following air pollution quality control manual (http://www.me.go.kr/home/web/index.do?menuId=10259). We used daily mean values.

References

http://nhiss.nhis.or.kr/.

Lee, J., J. S. Lee, S. H. Park, S. A. Shin, and K. Kim. 2017. 'Cohort Profile: The National Health Insurance Service-National Sample Cohort (NHIS-NSC), South Korea', *Int J Epidemiol*, 46: e15.

Song, S. O., C. H. Jung, Y. D. Song, C. Y. Park, H. S. Kwon, B. S. Cha, J. Y. Park, K. U. Lee, K. S. Ko, and B. W. Lee. 2014. 'Background and data configuration process of a nationwide population-based study using the korean national health insurance system', *Diabetes Metab J*, 38: 395-403.

Varini, M., L. Rinaldi, L. Bonetti, F. Neri, F. Santamaria, G. Stringari, and C. Caffarelli. 2014. 'Hypereosinophilia in a boy with asthma and Varicella Zoster Virus infection', *Acta Biomed*, 85: 64-7.

Table S2 Subgroup analyses of crude and adjusted odd ratios (95% confidence interval, CI) of the meteorological and pollution matter (mean of 30 days before index date) for Meniere's disease according to age, sex, income, and region of residence

Characteristics	Odds ratio for Meniere's disease (95% CI)	
	Model 2 †‡	P-value
Age < 60 years old (n = 15,035)		
Relative humidity for 30 days (%)	1.01 (1.00-1.01)	0.027*
Ambient atmospheric pressure for 30 days (hPa)	1.01 (1.01-1.02)	0.001*
O_3 for 30 days (0.01 ppm)	1.23 (1.16-1.30)	<0.001*
PM_{10} for 30 days (10 μg/m^3)	0.95 (0.92-0.99)	0.007*
Age ≥ 60 years old (n = 23,590)		
Temperature range for 30 days (˚C)	0.94 (0.91-0.97)	<0.001*
Ambient atmospheric pressure for 30 days (hPa)	1.01 (1.01-1.02)	<0.001*
Sunshine duration for 30 days (hr)	1.06 (1.01-1.10)	0.009*
SO_2 for 30 days (0.01 ppm)	0.61 (0.44-0.85)	0.003*
O_3 for 30 days (0.01 ppm)	1.30 (1.23-1.38)	<0.001*
CO for 30 days (ppm)	3.76 (2.43-5.82)	<0.001*
PM_{10} for 30 days (10 μg/m^3)	0.93 (0.89-0.96)	<0.001*
Males (n = 13,740)		
Ambient atmospheric pressure for 30 days (hPa)	1.01 (1.00-1.02)	0.002*
SO_2 for 30 days (0.01 ppm)	0.61 (0.41-0.91)	0.014*
O_3 for 30 days (0.01 ppm)	1.33 (1.24-1.43)	<0.001*

CO for 30 days (ppm)	3.12 (1.80-5.41)	<0.001*
PM_{10} for 30 days (10 μg/m^3)	0.93 (0.89-0.97)	0.001*
Females (n = 24,885)		
Temperature range for 30 days (˚C)	0.96 (0.93-0.98)	<0.001*
Ambient atmospheric pressure for 30 days (hPa)	1.01 (1.01-1.02)	<0.001*
SO_2 for 30 days (0.01 ppm)	0.68 (0.51-0.92)	0.011*
O_3 for 30 days (0.01 ppm)	1.28 (1.22-1.36)	<0.001*
CO for 30 days (ppm)	2.45 (1.63-3.67)	<0.001*
PM_{10} for 30 days (10 μg/m^3)	0.94 (0.91-0.97)	<0.001*
Low income (n = 17,490)		
Relative humidity for 30 days (%)	1.01 (1.00-1.02)	0.002*
Ambient atmospheric pressure for 30 days (hPa)	1.01 (1.01-1.02)	<0.001*
SO_2 for 30 days (0.01 ppm)	0.69 (0.49-0.97)	0.032*
O_3 for 30 days (0.01 ppm)	1.31 (1.22-1.39)	<0.001*
CO for 30 days (ppm)	3.25 (2.01-5.26)	<0.001*
PM_{10} for 30 days (10 μg/m^3)	0.91 (0.88-0.95)	<0.001*
High income (n = 21,135)		
Temperature range for 30 days (˚C)	0.92 (0.90-0.95)	<0.001*
Ambient atmospheric pressure for 30 days (hPa)	1.01 (1.01-1.02)	<0.001*
Sunshine duration for 30 days (hr)	1.09 (1.04-1.14)	<0.001*
SO_2 for 30 days (0.01 ppm)	0.55 (0.39-0.77)	0.001*

O_3 for 30 days (0.01 ppm)	1.28 (1.20-1.37)	<0.001*
CO for 30 days (ppm)	2.88 (1.84-4.53)	<0.001*
PM_{10} for 30 days (10 μg/m^3)	0.95 (0.92-0.99)	0.013*
Urban (n = 16,290)		
Temperature range for 30 days (˚C)	0.93 (0.90-0.98)	0.002*
Relative humidity for 30 days (%)	1.01 (1.01-1.02)	<0.001*
Ambient atmospheric pressure for 30 days (hPa)	1.03 (1.02-1.04)	<0.001*
Sunshine duration for 30 days (hr)	1.11 (1.06-1.16)	<0.001*
SO_2 for 30 days (0.01 ppm)	0.18 (0.13-0.27)	<0.001*
O_3 for 30 days (0.01 ppm)	1.38 (1.26-1.50)	<0.001*
CO for 30 days (ppm)	6.63 (3.97-11.05)	<0.001*
Rural (n = 22,335)		
Temperature range for 30 days (˚C)	0.90 (0.87-0.93)	<0.001*
Sunshine duration for 30 days (hr)	1.10 (1.05-1.16)	<0.001*
SO_2 for 30 days (0.01 ppm)	1.76 (1.34-2.31)	<0.001*
NO_2 for 30 days (0.1 ppm)	3.13 (1.32-7.43)	0.010*
O_3 for 30 days (0.01 ppm)	1.23 (1.16-1.30)	<0.001*
PM_{10} for 30 days (10 μg/m^3)	0.93 (0.90-0.97)	0.001*

Abbreviations: CCI, Charlson comorbidity index; DBP, diastolic blood pressure; SBP, systolic blood pressure

* Conditional logistic regression model, Significance at $P < 0.05$

† Stratified model for age, sex, income, and region of residence

‡ A model 2 was adjusted for total cholesterol, SBP, DBP, fasting blood glucose, obesity, smoking status, alcohol consumption, CCI score, benign paroxysmal vertigo, vestibular neuronitis, other peripheral vertigo, temperature range, relative humidity, ambient atmospheric pressure, sunshine duration, SO_2, NO_2, O_3, CO, and PM_{10} using forward selection method.

Table S3 Subgroup analyses of crude and adjusted odd ratios (95% confidence interval, CI) of the meteorological and pollution matter (mean of 180 days before index date) for Meniere's disease according to age, sex, income, and region of residence

Characteristics	Odds ratio for Meniere's disease (95% CI)	
	Model 2 †‡	P-value
Age < 60 years old (n = 15,035)		
Temperature range for 180 days (˚C)	0.94 (0.89-0.99)	0.011*
Ambient atmospheric pressure for 180 days (hPa)	1.02 (1.01-1.03)	<0.001*
Sunshine duration for 180 days (hr)	1.17 (1.07-1.28)	<0.001*
SO_2 for 180 days (0.01 ppm)	0.46 (0.28-0.73)	0.001*
O_3 for 180 days (0.01 ppm)	1.28 (1.15-1.42)	<0.001*
CO for 180 days (ppm)	3.82 (2.04-7.16)	<0.001*
PM_{10} for 180 days (10 μg/m^3)	0.87 (0.82-0.93)	<0.001*
Age ≥ 60 years old (n = 23,590)		
Temperature range for 180 days (˚C)	0.94 (0.91-0.98)	0.005*
Ambient atmospheric pressure for 180 days (hPa)	1.02 (1.01-1.03)	<0.001*
Sunshine duration for 180 days (hr)	1.18 (1.10-1.27)	<0.001*
SO_2 for 180 days (0.01 ppm)	0.52 (0.35-0.76)	0.001*
O_3 for 180 days (0.01 ppm)	1.23 (1.13-1.34)	<0.001*
CO for 180 days (ppm)	4.68 (2.75-7.98)	<0.001*
PM_{10} for 180 days (10 μg/m^3)	0.85 (0.81-0.90)	<0.001*
Males (n = 13,740)		
Temperature range for 180 days (˚C)	0.90 (0.86-0.95)	<0.001*

Ambient atmospheric pressure for 180 days (hPa)	1.02 (1.02-1.03)	<0.001*
Sunshine duration for 180 days (hr)	1.27 (1.16-1.39)	<0.001*
SO_2 for 180 days (0.01 ppm)	0.35 (0.21-0.59)	<0.001*
O_3 for 180 days (0.01 ppm)	1.26 (1.13-1.40)	<0.001*
CO for 180 days (ppm)	7.87 (3.90-15.90)	<0.001*
PM_{10} for 180 days (10 μg/m^3)	0.87 (0.81-0.93)	<0.001*
Females (n = 24,885)		
Temperature range for 180 days (˚C)	0.96 (0.92-1.00)	0.049*
Ambient atmospheric pressure for 180 days (hPa)	1.02 (1.01-1.02)	<0.001*
Sunshine duration for 180 days (hr)	1.11 (1.04-1.19)	0.002*
SO_2 for 180 days (0.01 ppm)	0.61 (0.42-0.88)	0.009*
O_3 for 180 days (0.01 ppm)	1.24 (1.15-1.34)	<0.001*
CO for 180 days (ppm)	3.06 (1.86-5.03)	<0.001*
PM_{10} for 180 days (10 μg/m^3)	0.86 (0.82-0.91)	<0.001*
Low income (n = 17,490)		
Ambient atmospheric pressure for 180 days (hPa)	1.02 (1.01-1.03)	<0.001*
SO_2 for 180 days (0.01 ppm)	0.64 (0.43-0.97)	0.033*
NO_2 for 180 days (0.1 ppm)	3.73 (1.48-9.37)	0.005*
O_3 for 180 days (0.01 ppm)	1.45 (1.31-1.60)	<0.001*
CO for 180 days (ppm)	3.59 (2.02-6.36)	<0.001*
PM_{10} for 180 days (10 μg/m^3)	0.79 (0.74-0.84)	<0.001*

High income (n = 21,135)		
Temperature range for 180 days (˚C)	0.91 (0.87-0.95)	<0.001*
Ambient atmospheric pressure for 180 days (hPa)	1.02 (1.01-1.03)	<0.001*
Sunshine duration for 180 days (hr)	1.28 (1.19-1.38)	<0.001*
SO_2 for 180 days (0.01 ppm)	0.40 (0.27-0.61)	<0.001*
O_3 for 180 days (0.01 ppm)	1.21 (1.11-1.32)	<0.001*
CO for 180 days (ppm)	4.78 (2.77-8.24)	<0.001*
PM_{10} for 180 days (10 μg/m^3)	0.89 (0.84-0.94)	<0.001*
Urban (n = 16,290)		
Temperature range for 180 days (˚C)	0.89 (0.84-0.95)	<0.001*
Relative humidity for 180 days (%)	1.02 (1.01-1.03)	0.001*
Ambient atmospheric pressure for 180 days (hPa)	1.07 (1.05-1.09)	<0.001*
Sunshine duration for 180 days (hr)	1.25 (1.15-1.36)	<0.001*
SO_2 for 180 days (0.01 ppm)	0.09 (0.05-0.15)	<0.001*
NO_2 for 180 days (0.1 ppm)	4.61 (1.57-13.56)	0.005*
O_3 for 180 days (0.01 ppm)	1.65 (1.41-1.93)	<0.001*
CO for 180 days (ppm)	8.80 (4.27-18.12)	<0.001*
PM_{10} for 180 days (10 μg/m^3)	0.93 (0.86-1.00)	0.039*
Rural (n = 22,335)		
Temperature range for 180 days (˚C)	0.82 (0.78-0.87)	<0.001*
Relative humidity for 180 days (%)	0.98 (0.97-0.99)	<0.001*
Sunshine duration for 180 days (hr)	1.31 (1.21-1.41)	<0.001*

SO_2 for 180 days (0.01 ppm)	2.04 (1.35-3.06)	0.001*
NO_2 for 180 days (0.1 ppm)	3.96 (1.62-9.73)	0.003*
CO for 180 days (ppm)	0.52 (0.30-0.89)	0.018*
PM_{10} for 180 days (10 μg/m^3)	0.87 (0.81-0.93)	<0.001*

Abbreviations: CCI, Charlson comorbidity index; DBP, diastolic blood pressure; SBP, systolic blood pressure

* Conditional logistic regression model, Significance at $P < 0.05$

† Stratified model for age, sex, income, and region of residence

‡ A model 2 was adjusted for total cholesterol, SBP, DBP, fasting blood glucose, obesity, smoking status, alcohol consumption, CCI score, benign paroxysmal vertigo, vestibular neuronitis, other peripheral vertigo, temperature range, relative humidity, ambient atmospheric pressure, sunshine duration, SO_2, NO_2, O_3, CO, and PM_{10} using forward selection method.

Table S4 Crude and adjusted odd ratios (95% confidence interval, CI) of the meteorological and pollution matter (mean of 7 days before index date) for Meniere's disease

Characteristics	Odds ratio for Meniere's disease (95% CI)					
	Crude †	P-value	Model 1 †‡	P-value	Model 2 †§	P-value
Mean temperature for 7 days (˚C)	1.00 (1.00-1.01)	0.007*	1.00 (1.00-1.01)	0.058		
Highest temperature for 7 days (˚C)	1.00 (1.00-1.01)	0.010*	1.00 (1.00-1.01)	0.082		
Lowest temperature for 7 days (˚C)	1.00 (1.00-1.01)	0.005*	1.00 (1.00-1.01)	0.048*		
Temperature range for 7 days (˚C)	0.99 (0.98-1.00)	0.134	0.99 (0.98-1.00)	0.153		
Relative humidity for 7 days (%)	1.01 (1.00-1.01)	<0.001*	1.01 (1.00-1.01)	<0.001*	1.01 (1.01-1.01)	<0.001*
Ambient atmospheric pressure for 7 days (hPa)	1.00 (0.99-1.00)	0.004*	1.00 (0.99-1.00)	0.018*	1.01 (1.01-1.01)	<0.001*
Sunshine duration for 7 days (hr)	1.00 (0.99-1.01)	0.797	1.01 (0.99-1.02)	0.353		
Rainfall for 7 days (mm)	1.00 (1.00-1.01)	0.681	1.00 (1.00-1.01)	0.762		
SO_2 for 7 days (0.01 ppm)	0.67 (0.59-0.77)	<0.001*	0.69 (0.59-0.79)	<0.001*	0.69 (0.56-0.85)	<0.001*
NO_2 for 7 days (0.1 ppm)	0.31 (0.23-0.41)	<0.001*	0.44 (0.32-0.60)	<0.001*		
O_3 for 7 days (0.01 ppm)	1.14 (1.11-1.17)	<0.001*	1.14 (1.11-1.17)	<0.001*	1.22 (1.18-1.27)	<0.001*
CO for 7 days (ppm)	0.75 (0.64-0.87)	<0.001*	0.74 (0.62-0.88)	0.001*	1.89 (1.44-2.49)	<0.001*

PM_{10} for 7 days (10 μg/m^3)	0.98 (0.96-0.99)	<0.001*	0.98 (0.97-0.99)	0.005*	0.97 (0.96-0.99)	0.005*

Abbreviations: CCI, Charlson comorbidity index; DBP, diastolic blood pressure; SBP, systolic blood pressure

* Conditional logistic regression model, Significance at $P < 0.05$

† Stratified model for age, sex, income, and region of residence

‡ A model 1 was adjusted for total cholesterol, SBP, DBP, fasting blood glucose, obesity, smoking status, alcohol consumption, and CCI score.

§ A model 2 was adjusted for total cholesterol, SBP, DBP, fasting blood glucose, obesity, smoking status, alcohol consumption, CCI score, benign paroxysmal vertigo, vestibular neuronitis, other peripheral vertigo, temperature range, relative humidity, ambient atmospheric pressure, sunshine duration, SO_2, NO_2, O_3, CO, and PM_{10} using forward selection method.

Table S5 Crude and adjusted odd ratios (95% confidence interval, CI) of the meteorological and pollution matter (mean of 90 days before index date) for Meniere's disease

Characteristics	Odds ratio for Meniere's disease (95% CI)					
	Crude †	P-value	Model 1 †‡	P-value	Model 2 †§	P-value
Mean temperature for 90 days (˚C)	1.00 (1.00-1.00)	0.521	1.00 (1.00-1.00)	0.229		
Highest temperature for 90 days (˚C)	1.00 (1.00-1.00)	0.450	1.00 (0.99-1.00)	0.168		
Lowest temperature for 90 days (˚C)	1.00 (1.00-1.00)	0.588	1.00 (1.00-1.00)	0.299		
Temperature range for 90 days (˚C)	0.99 (0.97-1.01)	0.296	0.99 (0.97-1.00)	0.107	0.93 (0.91-0.96)	<0.001*
Relative humidity for 90 days (%)	1.00 (1.00-1.01)	0.160	1.00 (1.00-1.01)	0.253	1.01 (1.00-1.01)	0.036*
Ambient atmospheric pressure for 90 days (hPa)	1.00 (1.00-1.00)	0.883	1.00 (1.00-1.00)	0.767	1.02 (1.01-1.02)	<0.001*
Sunshine duration for 90 days (hr)	1.08 (1.05-1.11)	<0.001*	1.09 (1.06-1.13)	<0.001*	1.14 (1.09-1.19)	<0.001*
Rainfall for 90 days (mm)	0.98 (0.97-0.99)	<0.001*	0.98 (0.97-0.99)	<0.001*		
SO_2 for 90 days (0.01 ppm)	0.71 (0.60-0.83)	<0.001*	0.73 (0.62-0.87)	0.001*	0.54 (0.41-0.72)	<0.001*
NO_2 for 90 days (0.1 ppm)	0.24 (0.18-0.34)	<0.001*	0.38 (0.26-0.54)	<0.001*		
O_3 for 90 days (0.01 ppm)	1.17 (1.13-1.21)	<0.001*	1.16 (1.12-1.21)	<0.001*	1.30 (1.23-1.37)	<0.001*
CO for 90 days (ppm)	0.74 (0.61-0.90)	0.002*	0.75 (0.61-0.93)	0.007*	4.57 (3.15-6.62)	<0.001*

PM_{10} for 90 days (10 μg/m^3)	0.94 (0.92-0.96)	<0.001*	0.94 (0.92-0.96)	<0.001*	0.91 (0.88-0.94)	<0.001*

Abbreviations: CCI, Charlson comorbidity index; DBP, diastolic blood pressure; SBP, systolic blood pressure

* Conditional logistic regression model, Significance at $P < 0.05$

† Stratified model for age, sex, income, and region of residence

‡ A model 1 was adjusted for total cholesterol, SBP, DBP, fasting blood glucose, obesity, smoking status, alcohol consumption, and CCI score.

§ A model 2 was adjusted for total cholesterol, SBP, DBP, fasting blood glucose, obesity, smoking status, alcohol consumption, CCI score, benign paroxysmal vertigo, vestibular neuronitis, other peripheral vertigo, temperature range, relative humidity, ambient atmospheric pressure, sunshine duration, SO_2, NO_2, O_3, CO, and PM_{10} using forward selection method.

Table S6 Subgroup analyses of crude and adjusted odd ratios (95% confidence interval, CI) of the meteorological and pollution matter (mean of 7 days before index date) for Meniere's disease according to age, sex, income, and region of residence

Characteristics	Odds ratio for Meniere's disease (95% CI)	
	Model 2 †‡	P-value
Age < 60 years old (n = 15,035)		
Relative humidity for 7 days (%)	1.01 (1.01-1.01)	<0.001*
Ambient atmospheric pressure for 7 days (hPa)	1.01 (1.01-1.02)	0.001*
SO_2 for 7 days (0.01 ppm)	0.63 (0.46-0.87)	0.005*
O_3 for 7 days (0.01 ppm)	1.21 (1.14-1.28)	<0.001*
CO for 7 days (ppm)	1.50 (1.01-2.24)	0.045*
Age ≥ 60 years old (n = 23,590)		
Relative humidity for 7 days (%)	1.01 (1.00-1.01)	<0.001*
Ambient atmospheric pressure for 7 days (hPa)	1.01 (1.00-1.01)	0.008*
O_3 for 7 days (0.01 ppm)	1.17 (1.13-1.22)	<0.001*
Males (n = 13,740)		
Relative humidity for 7 days (%)	1.01 (1.00-1.01)	0.001*
Ambient atmospheric pressure for 7 days (hPa)	1.01 (1.00-1.02)	0.008*
SO_2 for 7 days (0.01 ppm)	0.61 (0.44-0.86)	0.005*
O_3 for 7 days (0.01 ppm)	1.23 (1.16-1.31)	<0.001*
CO for 7 days (ppm)	2.08 (1.35-3.19)	0.001*
Females (n = 24,885)		

Temperature range for 7 days (˚C)	0.98 (0.96-1.00)	0.022*
Relative humidity for 7 days (%)	1.01 (1.00-1.01)	0.027*
Ambient atmospheric pressure for 7 days (hPa)	1.01 (1.00-1.02)	0.001*
SO_2 for 7 days (0.01 ppm)	0.68 (0.53-0.89)	0.004*
O_3 for 7 days (0.01 ppm)	1.22 (1.16-1.28)	<0.001*
CO for 7 days (ppm)	1.82 (1.29-2.57)	0.001*
PM_{10} for 7 days (10 μg/m^3)	0.97 (0.95-1.00)	0.031*
Low income (n = 17,490)		
Relative humidity for 7 days (%)	1.01 (1.01-1.02)	<0.001*
Ambient atmospheric pressure for 7 days (hPa)	1.01 (1.00-1.02)	0.002*
O_3 for 7 days (0.01 ppm)	1.17 (1.12-1.23)	<0.001*
High income (n = 21,135)		
Relative humidity for 7 days (%)	1.01 (1.00-1.01)	0.002*
Ambient atmospheric pressure for 7 days (hPa)	1.01 (1.00-1.02)	0.005*
SO_2 for 7 days (0.01 ppm)	0.65 (0.49-0.85)	0.002*
O_3 for 7 days (0.01 ppm)	1.21 (1.15-1.27)	<0.001*
CO for 7 days (ppm)	1.49 (1.06-2.09)	0.021*
Urban (n = 16,290)		
Relative humidity for 7 days (%)	1.01 (1.00-1.01)	0.001*
Ambient atmospheric pressure for 7 days (hPa)	1.02 (1.01-1.03)	<0.001*

SO_2 for 7 days (0.01 ppm)	0.29 (0.22-0.38)	<0.001*
O_3 for 7 days (0.01 ppm)	1.28 (1.21-1.36)	<0.001*
CO for 7 days (ppm)	3.19 (2.19-4.63)	<0.001*
Rural (n = 22,335)		
Temperature range for 7 days (˚C)	0.95 (0.93-0.97)	<0.001*
Relative humidity for 7 days (%)	1.01 (1.00-1.01)	0.006*
Sunshine duration for 7 days (hr)	1.04 (1.01-1.07)	0.009*
SO_2 for 7 days (0.01 ppm)	1.56 (1.26-1.94)	<0.001*
O_3 for 7 days (0.01 ppm)	1.17 (1.12-1.22)	<0.001*

Abbreviations: CCI, Charlson comorbidity index; DBP, diastolic blood pressure; SBP, systolic blood pressure

* Conditional logistic regression model, Significance at $P < 0.05$

† Stratified model for age, sex, income, and region of residence

‡ A model 2 was adjusted for total cholesterol, SBP, DBP, fasting blood glucose, obesity, smoking status, alcohol consumption, CCI score, benign paroxysmal vertigo, vestibular neuronitis, other peripheral vertigo, temperature range, relative humidity, ambient atmospheric pressure, sunshine duration, SO_2, NO_2, O_3, CO, and PM_{10} using forward selection method.

Table S7 Subgroup analyses of crude and adjusted odd ratios (95% confidence interval, CI) of the meteorological and pollution matter (mean of 90 days before index date) for Meniere's disease according to age, sex, income, and region of residence

Characteristics	Odds ratio for Meniere's disease (95% CI)	
	Model 2 †‡	P-value
Age < 60 years old (n = 15,035)		
Temperature range for 90 days (˚C)	0.92 (0.88-0.97)	0.001*
Relative humidity for 90 days (%)	1.01 (1.00-1.02)	0.019*
Ambient atmospheric pressure for 90 days (hPa)	1.02 (1.01-1.03)	<0.001*
Sunshine duration for 90 days (hr)	1.17 (1.09-1.26)	<0.001*
SO_2 for 90 days (0.01 ppm)	0.43 (0.28-0.66)	<0.001*
O_3 for 90 days (0.01 ppm)	1.29 (1.18-1.40)	<0.001*
CO for 90 days (ppm)	4.16 (2.33-7.43)	<0.001*
PM_{10} for 90 days (10 μg/m^3)	0.94 (0.89-0.99)	0.018*
Age ≥ 60 years old (n = 23,590)		
Ambient atmospheric pressure for 90 days (hPa)	1.02 (1.01-1.02)	<0.001*
O_3 for 90 days (0.01 ppm)	1.34 (1.26-1.43)	<0.001*
CO for 90 days (ppm)	3.31 (2.16-5.08)	<0.001*
PM_{10} for 90 days (10 μg/m^3)	0.87 (0.83-0.90)	<0.001*
Males (n = 13,740)		
Temperature range for 90 days (˚C)	0.90 (0.86-0.94)	<0.001*
Ambient atmospheric pressure for 90 days (hPa)	1.02 (1.01-1.03)	<0.001*

Sunshine duration for 90 days (hr)	1.24 (1.15-1.34)	<0.001*
SO_2 for 90 days (0.01 ppm)	0.33 (0.20-0.52)	<0.001*
O_3 for 90 days (0.01 ppm)	1.30 (1.19-1.42)	<0.001*
CO for 90 days (ppm)	6.78 (3.56-12.93)	<0.001*
PM_{10} for 90 days (10 μg/m^3)	0.93 (0.88-0.99)	0.016*
Females (n = 24,885)		
Temperature range for 90 days (˚C)	0.97 (0.94-1.00)	0.020*
Ambient atmospheric pressure for 90 days (hPa)	1.01 (1.01-1.02)	<0.001*
O_3 for 90 days (0.01 ppm)	1.31 (1.23-1.39)	<0.001*
CO for 90 days (ppm)	2.64 (1.76-3.96)	<0.001*
PM_{10} for 90 days (10 μg/m^3)	0.89 (0.85-0.92)	<0.001*
Low income (n = 17,490)		
Relative humidity for 90 days (%)	1.01 (1.00-1.01)	0.026*
Ambient atmospheric pressure for 90 days (hPa)	1.02 (1.01-1.02)	<0.001*
O_3 for 90 days (0.01 ppm)	1.35 (1.26-1.46)	<0.001*
CO for 90 days (ppm)	3.56 (2.18-5.82)	<0.001*
PM_{10} for 90 days (10 μg/m^3)	0.86 (0.82-0.90)	<0.001*
High income (n = 21,135)		
Temperature range for 90 days (˚C)	0.90 (0.87-0.93)	<0.001*
Ambient atmospheric pressure for 90 days (hPa)	1.02 (1.01-1.02)	<0.001*
Sunshine duration for 90 days (hr)	1.18 (1.11-1.26)	<0.001*

SO_2 for 90 days (0.01 ppm)	0.47 (0.32-0.68)	<0.001*
O_3 for 90 days (0.01 ppm)	1.28 (1.19-1.38)	<0.001*
CO for 90 days (ppm)	4.29 (2.61-7.04)	<0.001*
PM_{10} for 90 days (10 μg/m^3)	0.94 (0.90-0.98)	0.005*
Urban (n = 16,290)		
Temperature range for 90 days (˚C)	0.90 (0.86-0.95)	<0.001*
Relative humidity for 90 days (%)	1.01 (1.00-1.02)	0.010*
Ambient atmospheric pressure for 90 days (hPa)	1.04 (1.02-1.05)	<0.001*
Sunshine duration for 90 days (hr)	1.19 (1.11-1.27)	<0.001*
SO_2 for 90 days (0.01 ppm)	0.15 (0.09-0.25)	<0.001*
O_3 for 90 days (0.01 ppm)	1.48 (1.33-1.64)	<0.001*
CO for 90 days (ppm)	12.17 (6.60-22.42)	<0.001*
PM_{10} for 90 days (10 μg/m^3)	0.94 (0.89-1.00)	0.034*
Rural (n = 22,335)		
Temperature range for 90 days (˚C)	0.85 (0.82-0.88)	<0.001*
Sunshine duration for 90 days (hr)	1.25 (1.16-1.34)	<0.001*
SO_2 for 90 days (0.01 ppm)	1.78 (1.38-2.30)	<0.001*
O_3 for 90 days (0.01 ppm)	1.10 (1.04-1.17)	0.002*

Abbreviations: CCI, Charlson comorbidity index; DBP, diastolic blood pressure; SBP, systolic blood pressure

* Conditional logistic regression model, Significance at $P < 0.05$

† Stratified model for age, sex, income, and region of residence

‡ A model 2 was adjusted for total cholesterol, SBP, DBP, fasting blood glucose, obesity, smoking status, alcohol consumption, CCI score, benign paroxysmal vertigo, vestibular neuronitis, other peripheral vertigo, temperature range, relative humidity, ambient atmospheric pressure, sunshine duration, SO_2, NO_2, O_3, CO, and PM_{10} using forward selection method.

Table S8 The range of meteorological factors

Characteristics	Total participants	
	Meniere's disease (Minimum to Maximum)	Control (Minimum to Maximum)
Mean temperature for 30 days (˚C)	-7.9 to 30.0	-8.7 to 30.0
Highest temperature for 30 days (˚C)	-3.1 to 35.3	-3.4 to 35.4
Lowest temperature for 30 days (˚C)	-13.7 to 26.0	-14.9 to 26.1
Temperature range for 30 days (˚C)	4.1 to 15.8	4.1 to 15.6
Relative humidity for 30 days (%)	33.5 to 95.3	31.9 to 95.5
Ambient atmospheric pressure for 30 days (hPa)	978.0 to 1,022.6	978.1 to 1,022.8
Sunshine duration for 30 days (hr)	1.1 to 10.1	1.0 to 10.1
Rainfall for 30 days (mm)	2.1 to 39.6	1.6 to 44.7
SO_2 for 30 days (ppb)	1.2 to 13.7	1.1 to 14.8
NO_2 for 30 days (ppb)	2.9 to 85.2	2.8 to 98.1
O_3 for 30 days (ppb)	5.2 to 56.4	5.1 to 57.1
CO for 30 days (ppb)	191.0 to 1,494.6	143.7 to 1,494.6
PM_{10} for 30 days (μg/m^3)	18.4 to 116.8	18.3 to 123.7
Mean temperature for 180 days (˚C)	1.0 to 23.8	0.9 to 23.9
Highest temperature for 180 days (˚C)	5.3 to 29.1	4.8 to 29.2
Lowest temperature for 180 days (˚C)	-4.8 to 20.4	-4.9 to 20.8
Temperature range for 180 days (˚C)	5.3 to 12.8	5.2 to 12.7
Relative humidity for 180 days (%)	44.7 to 81.9	44.5 to 82.1

Ambient atmospheric pressure for 180 days (hPa)	983.2 to 1,017.6	982.8 to 1,017.7
Sunshine duration for 180 days (hr)	3.9 to 8.0	3.1 to 8.0
Rainfall for 180 days (mm)	3.9 to 14.7	3.8 to 16.0
SO_2 for 180 days (ppb)	1.6 to 10.5	1.6 to 11.8
NO_2 for 180 days (ppb)	5.8 to 46.5	5.9 to 46.7
O_3 for 180 days (ppb)	9.1 to 46.6	8.7 to 46.6
CO for 180 days (ppb)	268.0 to 1,010.4	265.5 to 1,010.2
PM_{10} for 180 days (μg/m^3)	27.5 to 82.1	26.9 to 82.9
